# Prognosis and Prognostic Factors of Serous Borderline Tumor-Micropapillary Variant: Retrospective Study of 200 Patients with Long-Term Follow-Up

**DOI:** 10.1155/2022/1655422

**Published:** 2022-10-10

**Authors:** Shuang-Zheng Jia, Hong-Wen Yao, Ning Li, Jun-Jun Yang, Yang Xiang, Shan Zheng, Jin-Hua Leng, Ling-Ying Wu

**Affiliations:** ^1^Department of Gynecologic Oncology, National Cancer Center /National Clinical Research Center for Cancer/Cancer Hospital, Chinese Academy of Medical Sciences and Peking Union Medical College, Beijing, China; ^2^Department of Obstetrics & Gynecology, Peking Union Medical College Hospital, Chinese Academy of Medical Sciences & Peking Union Medical College, Beijing, China; ^3^Department of Pathology, National Cancer Center/National Clinical Research Center for Cancer/Cancer Hospital, Chinese Academy of Medical Sciences and Peking Union Medical College, Beijing, China

## Abstract

**Objective:**

To determine the oncofertility outcomes and prognostic factors in a large series of serous borderline ovarian tumor-micropapillary variant (SBOT-M) with a long-term follow-up.

**Methods:**

Consecutive patients with SBOT-Ms treated from two affiliated hospitals of the Chinese Academy of Medical Sciences were retrospectively reviewed. Prognostic factors on invasive recurrence, disease-free survival (DFS), and overall survival were analyzed, and outcomes of patients treated with conservative and radical surgery were compared.

**Results:**

From 2000 to 2020, 200 patients were identified and followed. After a median follow-up of 68 months, 81 patients relapsed. In the multivariate analyses, younger age at diagnosis and conservative surgery that preserved fertility potential were independently associated with worse DFS (*p* = 0.018 and <0.001, respectively). Twenty-three patients experienced invasive recurrence, and seven died of progressive disease. Multivariate analysis showed that nulliparous and advanced FIGO stage were independently adversely associated with lethal recurrence (*p* = 0.022 and 0.029, respectively). Only advanced FIGO stage at diagnosis was associated with worse overall survival at univariate analysis (*p* = 0.02). Among 61 patients attempting conception, 37 achieved 44 pregnancies and resulted in 32 live births.

**Conclusions:**

In this series, patients with SBOT-M have an acceptable oncofertility outcomes. The use of conservative surgery was independently associated with worse DFS, but without an impact on neither invasive relapse nor on overall survival. Patients with advanced FIGO stages had a significantly higher risk of lethal recurrence and worse overall survival, suggesting that adequate staging surgery and intensive postoperative surveillance should be warranted.

## 1. Introduction

Serous borderline ovarian tumor-micropapillary variant (SBOT-M, also referred to as noninvasive low-grade serous carcinoma), the aggressive variant of SBOTs, makes up approximately one-quarter of all cases of SBOTs [1–5]. It was first described by Burks et al. [6] and Seidman and Kurman [7] in 1996 and is characterized by SBOTs with nonhierarchical branching architecture featuring micropapillary and/or cribriform patterns [8]. Compared to their typical counterparts, patients with SBOT-Ms are more commonly associated with extraovarian implant (particularly invasive implants), the presence of bilateral disease and areas of microinvasion, increased tumor recurrence, and higher mortality [9–11].

Given its recent introduction and specific features, however, very few data have specifically addressed the prognosis and feasibility of fertility preservation in patients with SBOT-Ms. Most available studies have focused mainly on the prognostic impact of the micropapillary pattern on SBOTs and documented poor disease-free survival (DFS) and high invasive evolution risk for women with SBOT-Ms [3, 4, 10–16]. Nevertheless, given the higher rate of concomitant extraovarian disease in SBOT-Ms, whether this risk is due to the intrinsic biology of micropapillary pattern or to the coexisting implants continues to fuel debate. In patients with advanced-stage disease, several studies have reported similar prognosis in SBOT with and without a micropapillary pattern [11, 15–19]. On the contrary, in the pooled analyses by Vasconcelos et al., patients with SBOT-Ms (regardless of stage) have a significantly higher rate of lethal recurrence than patients with advanced-stage SBOTs (implants of any type, regardless of the presence of SBOT-Ms or not) [5]. Regarding reproductive outcomes, only two retrospective series with small sample sizes (*n* = 8 and 15, retrospectively) have been published [17, 20].

Thus, the objective of current series was to describe the clinical characteristics and outcomes of patients with SBOT-Ms and to evaluate the safety of conservative surgery in selected patients. To our knowledge, this is the most extensive series of patients with SBOT-Ms that specifically dedicated to determine their prognostic factors with an extended follow-up.

## 2. Materials and Methods

### 2.1. Study Population

Consecutive patients with SBOT-Ms treated between January 2000 and June 2020 were identified retrospectively from two affiliated hospitals of the Chinese Academy of Medical Sciences. Data on demographics, clinicopathological findings, follow-up information, and fertility outcomes were retrieved from medical records or by telephone interview. Patients who were lost to follow-up within six months after initial surgeries were excluded. Institutional Review Board approval was obtained at both institutions, and verbal informed consent was obtained during follow-up visits or telephone interviews.

Pathology slides were reviewed by an experienced pathologist from each institution according to the 2014 WHO classification [8], and no centralized pathological review has been performed. When there was disagreement, the slides were rereviewed, and a consensus was reached via a discussion. SBOT-M is defined as SBOT with nonhierarchical branching papillae featuring either elongated filiform “micropapillae” (≥ 5 : 1 length to width ratio) or cribriform epithelial lining the cyst walls [6–8]. We classified extraovarian peritoneal implants as noninvasive or invasive based on the absence or presence of destructive stromal invasion of the underlying tissues [1, 21]. Ovarian tumors with stromal microinvasion were defined as the presence of stromal infiltration <10mm^2^ or < 5 mm. Surgical stage was determined using the 2014 FIGO classification system for ovarian cancer based on surgical and pathological findings [22].

### 2.2. Treatments and Follow-Up

The surgical treatment modality was determined after discussion with the individual based on the disease extent, the surgical teams, age of the patient, and fertility-preservation desire. Surgery consisted of either radical (bilateral salpingo-oophorectomy with or without hysterectomy) or conservative treatment (defined as salvage of the uterus and at least parts of one ovary). Staging quality was considered comprehensive when all peritoneal surfaces were carefully explored by cytology, random or oriented multiple biopsies, and omentectomy [23]. Adjuvant chemotherapy was decided by the treating physicians based on the pathological findings and date of the treatment.

Patients were followed up with a pelvic examination, ultrasound scan, and CA-125 evaluation every three months during the first year after surgery, then every six months for two years, and yearly thereafter.

### 2.3. Statistical Analyses

Two end-points were retained for the statistical analysis: (1) the rate of recurrence (purely borderline recurrence and as evolutive invasive disease) and (2) the rate of invasive recurrence in the form of invasive adenocarcinoma or invasive implants during follow-up [11, 14–16, 19, 24–26]. All recurrence was diagnosed radiographically or clinically and confirmed histologically. Disease-free survival (DFS) was calculated from the date of surgery to the date of first recurrence or the last follow-up. And invasive DFS was calculated as the interval from surgery to invasive recurrence (recurrence in the form of invasive adenocarcinoma or invasive implants) or last follow-up. Given the relatively indolent behaviors of SBOT-Ms and low-grade serous carcinoma, we defined overall survival (OS) as a secondary outcome, which was measured from surgery to disease-related death or last contact [14, 15, 19]. Survival analyses were conducted using the Kaplan–Meier method and compared with the log-rank test, and a Cox regression model with forward stepwise selection was used for multivariate analyses, including prognostic factors that were statistically significant in the univariate analysis. Concerning fertility outcomes, we defined pregnancy as visualization of a gestational sac with positive serum *β*-HCG levels. All statistical analyses were performed using SPSS 25.0 and GraphPad Prism 5.0, and a *p* value of <0.05 was considered significant.

## 3. Results

### 3.1. Patient Characteristics

During the study period, 222 patients with SBOT-Ms were identified, and 22 were excluded due to the lack of sufficient follow-up. Among 200 patients included in the current analysis, 122 had undergone conservative surgery, and 78 received radical therapy during their management. The demographics and tumor characteristics of the study cohort stratified by treatment modality are summarized and compared in Table 1.

The median age at the time of diagnosis was 32 years (range, 17-68 years), and patients who underwent conservative surgery were significantly younger and more likely to be nulliparous than those in the radical surgery group (p < 0.001 and <0.001, respectively). Two-thirds of the tumors were resected with the open approach (67.0%, *n* = 134/200) during their initial therapies, and complete staging surgery was performed in 127 patients (63.5%). After surgery, 54 patients (27.0%) received multiple different platinum-based chemotherapies due to extraovarian implants (20 invasive and 23 noninvasive), stromal microinvasion (*n* = 3), or unspecified reasons managed outside our hospitals (*n* = 8). The proportions of laparotomy approach, complete staging, and postoperative chemotherapy were significantly higher in the radical group (p < 0.001, *<*0.001, and <0.001, respectively).

Regarding pathological features, 137 patients (68.5%) had bilateral ovarian involvement, and 94 (47.0%) had extraovarian implants, including invasive and noninvasive implants, in 26 (13.0%) and 68 (34.0%) of affected patients, respectively. The proportions of patients with bilateral ovarian involvement (62.3% versus 78.2%, *p* = 0.018) and extraovarian implants (36.1% versus 64.1%, p < 0.001) were significantly lower in the conservative group.

### 3.2. Oncological Outcomes

After a median follow-up of 68 months (range, 6-240 months), 81 patients (40.5%) recurred in a delay from 3 to 140 months after their initial surgeries, including 73 (*n* = 73/122, 59.8%) in the conservative group and 8 (*n* = 8/78, 10.3%) in the radical group.  Table 2 shows the characteristics of patients with recurrence. Of the 81 patients with relapse, 72 were borderline at first recurrences, 34 had 2nd recurrences, and 13 experienced 3rd to 5th recurrences. In the radical group, the most common sites of recurrence were the peritoneum seeding (*n* = 4) and with bowel involvement (*n* = 4). In the conservative group, however, the most common sites of recurrence were the isolated ovary/ovaries (*n* = 47) and with peritoneal seeding (*n* = 26). Of these, 50 patients were salvaged with further conservative surgery.

**Figure 1:**
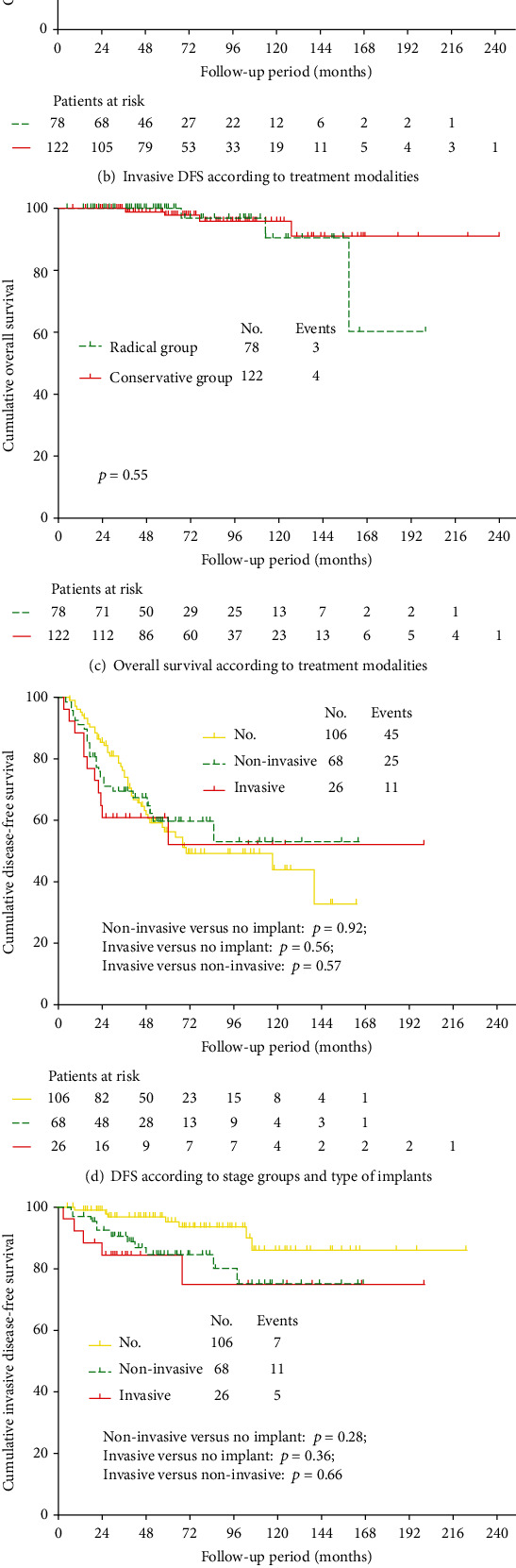
Survival curves in women with serous borderline ovarian tumor-micropapillary variants (SBOT-Ms).

The results of univariate analysis on DFS are summarized in Table 2. Univariate analysis of our data demonstrated that aged ≤35 years, nulliparous, incomplete staging surgery, not underwent lymphadenectomy, and conservative surgical extent (Figure 1(a)) were significantly associated with poorer DFS (*p* < 0.001, <0.001, =0.015, <0.001, and <0.001, respectively). As shown in Table 2, none of the pathological features was associated with DFS. After multivariate analysis, the use of conservative surgery and younger age at diagnosis was significantly associated with worse DFS (conservative versus radical, HR: 5.8, 95% CI: 2.6-12.8, *p* < 0.001; age ≤ 35 years versus >35 years, HR: 2.4, 95% CI: 1.2-4.9, *p* = 0.018, respectively).

Of the 81 patients with relapse, 23 (28.4%) developed invasive disease in a delay from 3 to 106 months after their initial surgeries, including 16 (*n* = 16/122, 13.1%) in the conservative group and seven (*n* = 7/78, 9.0%) in the radical group. Of these cases, 14 relapsed as low-grade serous carcinoma (LGSC), and nine recurred as invasive peritoneal disease. The 5-year invasive DFS rates in the conservative and radical surgery groups were 88% and 90%, respectively (*p* = 0.452) (Figure 1(b)). Univariate analysis showed that nulliparous, bilateral ovarian involvement, and advanced FIGO stage (Figure 1(e)) were significantly associated with the risk of invasive DFS (*p* = 0.009, 0.015, and 0.014, respectively), and multivariable analysis found that nulliparous and advanced FIGO stage were independently associated with the invasive evolution of the disease (nulliparous versus parous, HR: 3.5, 95% CI: 1.2-10.4, *p* = 0.022; advanced versus early stage, HR: 1.6, 95% CI: 1.1-2.6, *p* = 0.029, respectively) (Table 3). Patients with noninvasive and invasive implants showed similar invasive DFS (*p* = 0.66). Notably, among 36 patients with advanced-stage disease experiencing relapse, 44% (*n* = 16) were diagnosed with invasive recurrence. On the other hand, among 45 patients with stage I disease experiencing recurrence, one seven (16%) developed lethal recurrence.

At the time of analysis, seven patients (3.5%) had died of their disease at a range of 37 to 158 months following primary surgeries, including four (*n* = 4/122, 3.3%) in the conservative group and three (*n* = 3/78, 3.8%) in the radical group. The 5-year overall survival rates in the conservative and radical surgery groups were 97% and 97%, respectively. No significant difference in overall survival was found between different treatment modalities (*p* = 0.542) (Figure 1(c)). As shown in Table 4, only advanced FIGO stage at diagnosis was associated with worse OS at univariate analysis (*p* = 0.017) (Figure 1(f)).

### 3.3. Fertility Outcomes

Among 61 patients attempting conception, 37 (61%) achieved 44 pregnancies (32 live births, 5 induced abortions, 6 spontaneous miscarriages, and 1 ectopic pregnancy). Ten patients had undergone IVF-ET, achieving 5 pregnancies. At the time of analysis, among these patients, 10 experienced invasive recurrence, and one died of progressive disease.

## 4. Discussion

To our knowledge, this is the most extensive series available that explores the oncofertility outcomes of women with SBOT-Ms and their related predictors. Compared to radical management, the conservative procedure was associated with decreased DFS rates but not associated with invasive-specific or overall survival. Advanced FIGO stage at diagnosis was the strongest predictor of invasive evolution of the disease and worse OS, especially for those experiencing recurrence.

First, our findings support the feasibility of conservative therapy in young patients with SBOT-Ms and should be seriously discussed with these subjects. The 5-year DFS rate after conservative surgery in our series is 36.2%, which is significantly worse than that of the radical treatment group (88.7%, *p* < 0.001) (Figure 1(a)), as well as the probability reported for SBOT-Ms regardless of treatment modality, which varies from 61 to 86% [2, 17, 20]. However, 89% (*n* = 72/81) of our first recurrences were borderline lesions that could be cured readily by a second surgical procedure. Similar findings were also observed by Laurent et al. [20]; nearly 11 of 18 patients developed at least one recurrence at a median interval of 41 months, with only one patient experiencing lethal progression. Additionally, neither invasive DFS (Figure 1(b)) nor OS (Figure 1(c)) of patients undergoing conservative management was significantly different than that of women undergoing radical management (*p* = 0.452 and 0.542, respectively). Thus, women with SBOT-Ms can be safely treated with FSS, but a high rate of recurrence is expected.

Second, our work suggests an association between SBOT-Ms and lethal recurrence risk, especially for those with extraovarian implants. Nearly one-ninth of our patients (11.5%, *n* = 23/200) experienced invasive relapse, including seven patients (3.5%) who eventually succumbed to their disease. This rate of invasive progress is comparable to that described in the literature review and national cohort studies for SBOT-Ms (11-21%) [5, 10, 18] and significantly higher than the widely reported rate for typical SBOTs (2-8%) [14, 15, 19, 27]. Additionally, in a systematic review by Vasconcelos et al. [5], 18.9% (*n* = 59/314) and 12.4% (*n* = 78/632) of patients with SBOT-Ms (regardless of stage) and advanced-stage disease (implants of any type, regardless of the presence of SBOT-Ms or not), respectively, progressed to invasive disease (*p* < 0.0005). That is, patients with SBOT-Ms have a significantly higher rate of lethal recurrence than patients with advanced-stage SBOTs, and therefore, SBOT-Ms should be regarded as a high-risk factor for lethal recurrence, especially for those with peritoneal disease.

Several reasons may account for this. First, several high-risk features for lethal recurrence are highly prevalent in SBOT-M cases, namely, bilateral involvement, residual disease after surgery, advanced-stage disease, stromal microinvasion, and the presence of invasive implants, although the data are conflicting [5, 10–12, 14, 15, 18–20, 28, 29]. Second, sample bias might exist. Histologic review of LGSC slides revealed that 52% of LGSC cases had concurrent SBOT-Ms [30], implying that occult invasion might have been unsampled during the initial management of SBOT-Ms. Finally, molecular studies have demonstrated shared clonal and MAPK pathway mutations between SBOT-Ms and LGSCs [31], suggesting SBOT-M as a stepwise pattern from SBOT to invasive ovarian cancer. Thus, a thorough sampling with at least 2 sections/cm of maximum tumor diameter in both the primary SBOT-M tumors and extraovarian implants should be considered to rule out occult invasion [32, 33], and patients with SBOT-Ms would benefit from more aggressive staging surgery and intensive follow-up at an oncology center [23].

It is not surprising to identify younger age at diagnosis as an adverse predictor for recurrence. In our cohort, patients ≤35 years were more likely to be treated with conservative surgery (25.3% versus 82.4%, *p* < 0.001), to use the laparoscopic approach (40.0% versus 21.3%, *p* = 0.007), and less likely to undergone adequate staging surgery (54.4% versus 78.7%, *p* = 0.001), compared to those >35 years. As shown in Table 2, all these factors were significantly associated with DFS in either univariate or multivariate analysis. Similar findings were also observed in the national cohort of the AGO ROBOT study [34], as well as in stage I SBOTs with conservative treatment [13]. However, both studies were conducted among the borderline/serous borderline population, without separating data on SBOT-Ms. Notably, for the first time, nulliparous was identified as an independent prognostic factor for invasive evolution (nulliparous versus parous, HR: 3.5, 95% CI: 1.2-10.4, *p* = 0.022). Nevertheless, in this nulliparous subgroup of women, there was a trend towards a higher rate of younger age at diagnosis (82.4% versus 39.1%, *p* < 0.001), bilateral tumors (76.9% versus 58.7%, *p* = 0.006), advanced stage at diagnosis (52.8% versus 40.2%, *p* = 0.076), and inadequate staging surgery (43.5% versus 28.3%, *p* = 0.025), compared to those parous women. As observed previously, nearly one-third of patients with “apparent stage I” SBOT-Ms were upstaged after adequate staging surgery [29, 32], and incomplete staging has an unfavorable impact on recurrence [4, 14]. That is, more patients might be upstaged if rigorous staging surgery had been performed in this nulliparous cohort, whereas advanced stage was independent associated with lethal relapse (HR: 1.6, 95% CI: 1.1-2.6, *p* = 0.029). In fact, stratification analysis of our data according to tumor stage revealed that nulliparous status was negatively associated with malignant transformation only in early-stage group (*p* = 0.016), but not in advanced-stage group (*p* = 0.23).

Last and foremost, our results indicate the stage-specific risk of malignant transformation for SBOT-Ms patients with recurrence. Compared with stage I disease, patients with extraovarian implants doubled the risk of malignant transformation (HR: 1.6, 95% CI: 1.1-2.6, *p* = 0.029), especially for those experiencing recurrence. Among 36 patients with advanced-stage disease experiencing relapse, 44% (*n* = 16) were diagnosed with invasive carcinoma and had a subsequent impaired survival, which was significantly higher than those with stage I disease (*n* = 7/45, 16%). Similar findings were also reported by Uzan et al.; of 53 patients with stage II-III SBOT-Ms, 13 patients had relapsed, and six of them recurred as invasive disease [17], whereas of 18 patients with stage I, six had relapsed, and only one developed invasive disease [14]. Further supporting data were from the national Denmark cohort involving 80 SBOT-Ms (in whom 58 stage I); patients with advanced stage showed a trend toward increasing risk of developing serous carcinoma, although not statistical significance (HR = 2.6; 95% CI: 0.6–10.5; stage II-III versus I) [19]. Additionally, as reviewed by Vasconcelos *et al.*, of 157 SBOT-M patients with lethal recurrence, only four occurred in the absence of peritoneal disease spread [5]. Several studies have postulated the type of implants and number of implants, and intratumoral heterogeneity might account for this apparent stage-specific invasive evolution [4, 10, 16, 29, 33]. However, the results from different studies are inconsistent, and comparable invasive DFS and OS were observed between invasive and noninvasive implants associated within our series (*p* = 0.66 and.87, respectively). Nevertheless, adequate staging surgery with careful exploration of all peritoneal surfaces and prolonged follow-up of these patients are justified, and further studies that explored early diagnosis of recurrence, particularly for invasive recurrences that are still localized, are urgently needed [35].

Of note, our study has several limitations. First, selection bias might exist due to the nature of all retrospective studies, although the study population was retrieved consecutively. Patients with “higher risk” features might be more inclined to select a more radical approach, and as observed in our cohort, more patients with advanced stage and invasive implants underwent radical surgery. However, there was no significant difference in either invasive recurrence or overall survival among the different treatment modalities, which reinforced the feasibility of fertility preservation in women with SBOT-Ms. Second, as referral centers, approximately one-third of the patients in the cohort were initially treated at an outside hospital and then referred to our institutions after recurrence; thus, referral bias is another weakness. A third limitation of the current study is that only two-thirds of the cases underwent complete staging during their initial surgery, which might underestimate the influence of extraovarian implants on prognosis. As observed previously, nearly one-third of patients with “apparent stage I” SBOT-Ms were upstaged after accurate staging surgery [29, 32], and incomplete staging has an unfavorable impact on recurrence [4, 14]. Nonetheless, to our knowledge, the current series represents the largest published report to date that specifically addresses the oncofertility outcomes of women with SBOT-Ms.

In conclusion, our series suggests that conservative surgery is a safe and effective modality for the treatment of young women with SBOT-Ms, with an acceptable oncological outcome and promising pregnancy result. The risk for lethal recurrence is not rare, especially for those with extraovarian implants experiencing relapse. Further studies that explore the potential malignant transformation mechanisms and postoperative surveillance modality are warranted.

## Figures and Tables

**Table 1:** Demographic and clinical features of the patients with SBOT-Ms (N = 200).

Parameters		Overall cohort, *n* (%), *N* = 200	Radical group, *n* (%), *n* = 78	Conservative group, *n* (%), *n* = 122	*p* value
Age (median, range, years)		32 (17-68)	42 (20-68)	28 (17-42)	<.001
Age, years ^†^	≤ 35	125 (62.5)	22 (28.2)	103 (84.4)	<.001
	> 35	75 (37.5)	56 (71.8)	19 (15.6)	
Nulliparous	Yes	108 (54.0)	20 (25.6)	88 (72.1)	<.001
	No	92 (46.0)	58 (74.4)	34 (27.9)	
CA-125 (median, range, U/mL)		210 (5.6-25000.0)	284.3 (5.6-25000.0)	175 (7.1-12313)	.346
Referral	Yes	64 (32.0)	21 (26.9)	43 (35.2)	.218
	No	136 (68.0)	57 (73.1)	79 (64.8)	
Surgical approach	Laparotomy	134 (67.0)	69 (88.5)	65 (53.3)	<.001
	Laparoscopy	66 (33.0)	9 (11.5)	57 (46.7)	
Complete staging	Yes	127 (63.5)	74 (94.9)	53 (43.4)	<.001
	No	73 (36.5)	4 (5.1)	69 (56.6)	
Lymphadenectomy	No	140 (70.0)	30 (38.5)	110 (90.2)	<.001
	Yes	60 (30.0)	48 (61.5)	12 (9.8)	
Ovarian involvement	Unilateral	63 (31.5)	17 (21.8)	46 (37.7)	.018
	Bilateral	137 (68.5)	61 (78.2)	76 (62.3)	
FIGO stage	I	106 (53.0)	28 (35.9)	78 (63.9)	<.001
	II-IV	94 (47.0)	50 (64.1)	44 (36.1)	
Stromal microinvasion	No	164 (82.0)	55 (70.5)	109 (89.3)	.001
	Yes	36 (18.0)	23 (29.5)	13 (10.7)	
Type of implants (*n* = 94)	Noninvasive	68 (72.3)	34 (68.0)	34 (77.3)	.316
	Invasive	26 (27.7)	16 (32.0)	10 (22.7)	
Adjuvant chemotherapy	No	146 (73.0)	42 (46.2)	104 (85.2)	<.001
	Yes	54 (27.0)	36 (53.8)	18 (14.8)	

Values are *n* (%) unless stated otherwise. ^†^Based on X-tile analysis.

**Table 2:** Prognostic factors for recurrence in women with SBOT-Ms (*N* = 200).

Variables		Disease-free survival (DFS)				
		Univariate analysis			Multivariate analysis	
		N recur/total (%)	5-year DFS (%)	*p*	Hazard ratio (95% CI)	*p*
Age (years)	> 35	10/75 (13.3)	86	<.001	1	.018
	≤ 35	71/125 (56.8)	39		2.4 (1.2-4.9)	
Nulliparous	No	21/92 (22.8)	76	<.001	1	.152
	Yes	60/108 (55.6)	40		—	
Surgical approach	Laparotomy	49/134 (36.6)	62	.066		
	Laparoscopy	32/66 (48.5)	43			
Staging surgery	Yes	42/127 (33.1)	64	.015	1	.054
	No	39/73 (53.4)	44		—	
Lymphadenectomy	Yes	11/60 (18.3)	80	<.001	1	.281
	No	70/140 (50.0)	45		—	
Surgical extent	Radical	8/78 (10.3)	89	<.001	1	<.001
	Conservative	73/122 (59.8)	36		5.8 (2.6-12.8)	
Ovarian involvement	Unilateral	21/63 (33.3)	62	.128		
	Bilateral	60/137 (43.8)	54			
FIGO stage^†^	I	45/106 (42.5)	56	.904		
	II-IV	36/94 (38.3)	57			
Stromal microinvasion	No	71/164 (43.3)	54	.114		
	Yes	10/36 (27.8)	65			
Type of implants (*n* = 94)	Noninvasive	25/68 (36.8)	58	.566		
	Invasive	11/26 (42.3)	52			
Chemotherapy	No	58/146 (39.7)	57	.887		
	Yes	23/54 (42.6)	54			

^†^FIGO, International Federation of Gynecology and Obstetrics.

**Table 3:** Prognostic factors for invasive recurrence in women with SBOT-Ms (*N* = 200).

Variables		Invasive disease-free survival (DFS)				
		Univariate analysis			Multivariate analysis	
		N. lethal recur./total (%)	5-year invasive DFS (%)	*p* value	Hazard ratio (95% CI)	*p* value
Age (years)	≤ 35	18/125 (14.4)	87	.165		
	>35	5/75 (6.7)	93			
Nulliparous	No	4/92 (4.3)	95	.009	1	.022
	Yes	19/108 (17.6)	86		3.5 (1.2-10.4)	
Surgical approach	Laparotomy	17/134 (12.7)	88	.574		
	Laparoscopy	6/66 (9.1)	92			
Staging surgery	Yes	15/127 (11.8)	90	.774		
	No	8/73 (11.0)	89			
Lymphadenectomy	Yes	7/60 (11.7)	93	>.999		
	No	16/140 (11.4)	89			
Surgical extent	Radical	7/78 (9.0)	90	0.452		
	Conservative	16/122 (13.1)	88			
Ovarian involvement	Unilateral	2/63 (3.2)	96	.015	1	.112
	Bilateral	21/137 (15.3)	87		—	
FIGO stage^†^	I	7/106 (6.6)	95	.014	1	.029
	II-IV	16/94 (17.0)	83		1.6 (1.1-2.6)	
Stromal microinvasion	No	17/164 (10.4)	92	.221		
	Yes	6/36 (16.7)	77			
Type of implants (*n* = 94)	Noninvasive	11/68 (16.2)	83	.657		
	Invasive	5/26 (19.2)	77			
Chemotherapy	No	13/146 (8.9)	93	.101		
	Yes	10/54 (18.5)	80			

^†^FIGO, International Federation of Gynecology and Obstetrics.

**Table 4:** Prognostic factors for overall survival in women with SBOT-Ms (*N* = 200).

Variables		Overall survival (OS)		
		Univariate analysis		
		N. DOD/total (%)	5-year OS (%)	*p* value
Age (years)	≤ 35	4/125 (3.2)	97	.383
	>35	3/75 (4.0)	97	
Nulliparous	No	3/92 (3.3)	98	.786
	Yes	4/108 (3.7)	96	
Surgical approach	Laparotomy	5/134 (3.7)	97	.920
	Laparoscopy	2/66 (3.0)	97	
Staging surgery	Yes	5/127 (3.9)	97	.628
	No	2/73 (2.7)	98	
Lymphadenectomy	Yes	3/60 (5.0)	96	.460
	No	4/140 (2.9)	97	
Surgical extent	Radical	3/78 (3.8)	97	.542
	Conservative	4/122 (3.3)	97	
Ovarian involvement	Unilateral	1/63 (1.6)	96	.257
	Bilateral	6/137 (4.4)	97	
FIGO stage^†^	I	1/106 (0.9)	96	.017
	II-IV	6/94 (6.4)	97	
Stromal microinvasion	No	5/164 (3.0)	98	.355
	Yes	2/36 (5.6)	97	
Type of implants (*n* = 94)	Noninvasive	4/68 (5.9)	97	.867
	Invasive	2/26 (7.7)	90	
Chemotherapy	No	4/146 (2.7)	98	.389
	Yes	3/54 (5.6)	96	

^†^FIGO, International Federation of Gynecology and Obstetrics.

## Data Availability

Data is available on request by contacting jiashuangzheng@cicams.ac.cn.

## References

[1] Slomovitz B. M., Caputo T. A., Gretz H. F. (2002). A comparative analysis of 57 serous borderline tumors with and without a noninvasive micropapillary component. *The American Journal of Surgical Pathology*.

[2] Park J. Y., Kim D. Y., Kim J. H. (2011). Micropapillary pattern in serous borderline ovarian tumors: does it matter?. *Gynecologic Oncology*.

[3] Shih K. K., Zhou Q., Huh J. (2011). Risk factors for recurrence of ovarian borderline tumors. *Gynecologic Oncology*.

[4] du Bois A., Ewald-Riegler N., de Gregorio N. (2013). Borderline tumours of the ovary: a cohort study of the Arbeitsgmeinschaft Gynäkologische Onkologie (AGO) Study Group. *European Journal of Cancer*.

[5] Vasconcelos I., Darb-Esfahani S., Sehouli J. (2016). Serous and mucinous borderline ovarian tumours: differences in clinical presentation, high-risk histopathological features, and lethal recurrence rates. *BJOG: an International Journal of Obstetrics and Gynaecology*.

[6] Burks R. T., Sherman M. E., Kurman R. J. (1996). Micropapillary serous carcinoma of the ovary: a distinctive low-grade carcinoma related to serous borderline tumors. *The American Journal of Surgical Pathology*.

[7] Seidman J. D., Kurman R. J. (1996). Subclassification of serous borderline tumors of the ovary into benign and malignant types. A clinicopathologic study of 65 advanced stage cases. *The American Journal of Surgical Pathology*.

[8] Kurman R. J., Carcangiu M. L., Herrington C. S., Young R. H. (2014). *WHO Classification of Tumours of Female Reproductive Organs*.

[9] Fauvet R., Demblocque E., Morice P., Querleu D., Daraï E. (2012). Behavior of serous borderline ovarian tumors with and without micropapillary patterns: results of a French multicenter study. *Annals of Surgical Oncology*.

[10] Vang R., Hannibal C. G., Junge J., Frederiksen K., Kjaer S. K., Kurman R. J. (2017). Long-term behavior of serous borderline tumors subdivided into atypical proliferative tumors and noninvasive low-grade carcinomas: a population-based clinicopathologic study of 942 cases. *The American Journal of Surgical Pathology*.

[11] Longacre T. A., McKenney J. K., Tazelaar H. D., Kempson R. L., Hendrickson M. R. (2005). Ovarian serous tumors of low malignant potential (borderline tumors). *The American Journal of Surgical Pathology*.

[12] Prat J., De Nictolis M. (2002). Serous borderline tumors of the ovary: a long-term follow-up study of 137 cases, including 18 with a micropapillary pattern and 20 with microinvasion. *The American Journal of Surgical Pathology*.

[13] Uzan C., Muller E., Kane A. (2014). Prognostic factors for recurrence after conservative treatment in a series of 119 patients with stage I serous borderline tumors of the ovary. *Annals of Oncology: Official Journal of the European Society for Medical Oncology*.

[14] Uzan C., Nikpayam M., Ribassin-Majed L. (2014). Influence of histological subtypes on the risk of an invasive recurrence in a large series of stage I borderline ovarian tumor including 191 conservative treatments. *Annals of Oncology: Official Journal of the European Society for Medical Oncology*.

[15] Jia S. Z., Xiang Y., Yang J. J., Shi J. H., Jia C. W., Leng J. H. (2020). Oncofertility outcomes after fertility-sparing treatment of bilateral serous borderline ovarian tumors: results of a large retrospective study. *Human Reproduction*.

[16] Maria S., Faron M., Maulard A. (2020). Long term follow-up of a large series of stage-II/III atypical proliferative serous ovarian tumors. *Gynecologic Oncology*.

[17] Uzan C., Kane A., Rey A. (2011). Prognosis and prognostic factors of the micropapillary pattern in patients treated for stage II and III serous borderline tumors of the ovary. *The Oncologist*.

[18] Morice P., Uzan C., Fauvet R., Gouy S., Duvillard P., Darai E. (2012). Borderline ovarian tumour: pathological diagnostic dilemma and risk factors for invasive or lethal recurrence. *The Lancet. Oncology*.

[19] Hannibal C. G., Vang R., Junge J., Frederiksen K., Kurman R. J., Kjaer S. K. (2017). A nationwide study of ovarian serous borderline tumors in Denmark 1978-2002\. Risk of recurrence, and development of ovarian serous carcinoma. *Risk of Recurrence, and Development of Ovarian Serous Carcinoma, Gynecologic Oncology*.

[20] Laurent I., Uzan C., Gouy S., Pautier P., Duvillard P., Morice P. (2008). Results after conservative treatment of serous borderline tumors of the ovary with a micropapillary pattern. *Annals of Surgical Oncology*.

[21] Bell K. A., Smith Sehdev A. E., Kurman R. J. (2001). Refined diagnostic criteria for implants associated with ovarian atypical proliferative serous tumors (borderline) and micropapillary serous carcinomas. *The American Journal of Surgical Pathology*.

[22] Prat J., FIGO Committee on Gynecologic Oncology (2014). Staging classification for cancer of the ovary, fallopian tube, and peritoneum. *International Journal of Gynaecology and Obstetrics: The Official Organ of the International Federation of Gynaecology and Obstetrics*.

[23] Colombo N., Sessa C., du Bois A. (2019). ESMO-ESGO consensus conference recommendations on ovarian cancer: pathology and molecular biology, early and advanced stages, borderline tumours and recurrent disease^†^. *Annals of Oncology : Official Journal of the European Society for Medical Oncology*.

[24] Gouy S., Maria S., Faron M. (2021). Results after conservative surgery of stage II/III serous borderline ovarian tumors. *Annals of Surgical Oncology*.

[25] Uzan C., Kane A., Rey A., Gouy S., Duvillard P., Morice P. (2010). Outcomes after conservative treatment of advanced-stage serous borderline tumors of the ovary. *Annals of Oncology : Official Journal of the European Society for Medical Oncology*.

[26] Leary A., Petrella M. C., Pautier P. (2014). Adjuvant platinum-based chemotherapy for borderline serous ovarian tumors with invasive implants. *Gynecologic Oncology*.

[27] Zanetta G., Rota S., Chiari S., Bonazzi C., Bratina G., Mangioni C. (2001). Behavior of borderline tumors with particular interest to persistence, recurrence, and progression to invasive carcinoma: a prospective study. *Journal of Clinical Oncology : Official Journal of the American Society of Clinical Oncology*.

[28] Deavers M. T., Gershenson D. M., Tortolero-Luna G., Malpica A., Lu K. H., Silva E. G. (2002). Micropapillary and cribriform patterns in ovarian serous tumors of low malignant potential: a study of 99 advanced stage cases. *The American Journal of Surgical Pathology*.

[29] Bristow R. E., Gossett D. R., Shook D. R. (2002). Micropapillary serous ovarian carcinoma: surgical management and clinical outcome. *Gynecologic Oncology*.

[30] Ahn G., Folkins A. K., McKenney J. K., Longacre T. A. (2016). Low-grade serous carcinoma of the ovary: clinicopathologic analysis of 52 invasive cases and identification of a possible noninvasive intermediate lesion. *The American Journal of Surgical Pathology*.

[31] May T., Virtanen C., Sharma M. (2010). Low malignant potential tumors with micropapillary features are molecularly similar to low-grade serous carcinoma of the ovary. *Gynecologic Oncology*.

[32] Shih K. K., Garg K., Soslow R. A., Chi D. S., Abu-Rustum N. R., Barakat R. R. (2011). Accuracy of frozen section diagnosis of ovarian borderline tumor. *Gynecologic Oncology*.

[33] Seidman J. D., Savage J., Krishnan J., Vang R., Kurman R. J. (2020). Intratumoral heterogeneity accounts for apparent progression of noninvasive serous tumors to invasive low-grade serous carcinoma: a study of 30 low-grade serous tumors of the ovary in 18 patients with peritoneal carcinomatosis. *International Journal of Gynecological Pathology : Official Journal of the International Society of Gynecological Pathologists*.

[34] Trillsch F., Mahner S., Woelber L. (2014). Age-dependent differences in borderline ovarian tumours (BOT) regarding clinical characteristics and outcome: results from a sub-analysis of the Arbeitsgemeinschaft Gynaekologische Onkologie (AGO) ROBOT study. *Annals of Oncology : Official Journal of the European Society for Medical Oncology*.

[35] Uzan C., Kane A., Rey A. (2011). How to follow up advanced-stage borderline tumours? Mode of diagnosis of recurrence in a large series stage II-III serous borderline tumours of the ovary. *Annals of Oncology : Official Journal of the European Society for Medical Oncology*.

